# Emergency Departments, simulation and leadership

**DOI:** 10.1186/1757-7241-20-S2-P18

**Published:** 2012-04-16

**Authors:** Gunhild Kjaergaard-Andersen

**Affiliations:** 1 Medical Education Center Haderslev, The hospital of Soenderjylland, Denmark

## Background

By establishing Emergency Departments (ED) in Denmark, new leadership roles are defined, such as flowmasters and patient progress leaders. In order to prepare the ED staff for these assignments the Medical Education Center Haderslev and The Academy of Leadership in southern Denmark developed a three days course in Leadership.

## Methods

We accomplished three training courses with twelve participants attending course. The first two days were theoretical introductions and the third day was a practical exercise, where the theories and tools learned at the first two days were tested in a case-based full-scale simulation with debriefing afterwards. The simulation reflected different set-ups from a busy clinical day where the focus was on the management of the leadership rather than the clinical skills.

All three days were closed by a verbal and written evaluation of the outcome.

A questionnaire about the academic content, outcome, application to the clinically work in the departments etc graded from 1 to 6 was used and 6 was best.

## Results

The evaluations showed the importance of trying the leadership role using simulation because it uncovered the participants strengths and weaknesses.

The Simulation also enriched the discussion of the future challenges in the ED. Furthermore the exercises revealed that the flowmaster assignments was best handled when the participant acted in teams of two staff members, who had no clinical duties because this lead to quickly loss of overview.

The participants also realised the importance of clear and directly communication among the staff in order to create good patient flow in the ED daily practice in their own departments.

The students in the courses were specially selected and maybe more motivated which might influence the evaluations. The future students will be more randomly chosen.

## Conclusion

A three day course including theoretical aspects as well as simulation is suitable for training of leadership roles in Emergency Departments.

